# Efficacy and safety of Ashwagandha (*Withania somnifera*) root extract on stress and weight management in adults: a prospective, randomized, double-blind, placebo-controlled study

**DOI:** 10.25122/jml-2025-0147

**Published:** 2025-12

**Authors:** Ketan Pakhale, Rajshree Pakhale, Mayakalyani Srivathsan, Jayshree Langade, Deepak Langade

**Affiliations:** 1 Metabolic Syndrome, Metabol-Lifestyle Clinic for Metabolic Syndrome, Mumbai, Maharashtra, India; 2 Clinical Research, Clinsearch Healthcare Solutions, Thane, Maharashtra, India; 3 Pharmacology, D Y Patil University School of Medicine, Nerul, Navi Mumbai, Maharashtra, India

**Keywords:** stress, psychological, overweight, *Withania somnifera*, safety, AEs, adverse events, ANOVA, analysis of variance, ARE, Ashwagandha root extract, BMI, body mass index, CA2, carbonic anhydrase II, CA3, carbonic anhydrase III, CBC, complete blood count, CLIA, chemiluminescent immunoassay, CONSORT, Consolidated Standards of Reporting Trials, CTRI, Clinical Trials Registry of India, FCQ-T, Food Cravings Questionnaire-Trait, GLM, General Linear Model, HPA, hypothalamic-pituitary-adrenal axis, ICH-GCP, International Council for Harmonisation Good Clinical Practice, IEC, Institutional Ethics Committee, LFT, liver function tests, MCS, Mental Component Summary, PCS, Physical Component Summary, PL, placebo, PSS, Perceived Stress Scale, RCT, randomized controlled trial, SD, standard deviation, SSS, Subjective Satisfaction Scale, T3, triiodothyronine, T4, thyroxine, TEAEs, treatment-emergent adverse events

## Abstract

The relationship between excess body weight and stress significantly impacts overall health. The current study evaluated the efficacy and safety of Ashwagandha (*Withania somnifera*) root extract (ARE) in altering stress biomarkers and its role in weight management. In this prospective, randomized, double-blind, placebo-controlled study, 100 participants (age: 19-65 years) were randomized to receive either ARE 300 mg twice daily (*n* = 50) or an identical placebo (PL, *n* = 50) for 24 weeks. The primary efficacy endpoints were changes in body weight and body mass index (BMI). Secondary endpoints included evaluation of stress (Perceived Stress Scale, PSS), quality of life (Short Form-12 Quality of Life Scale), subjective satisfaction (Subjective Satisfaction Scale, SSS), and food cravings (Food Cravings Questionnaire-Trait, FCQ-T). Clinical safety was assessed based on treatment-emergent adverse events (TEAEs) and laboratory parameters, including complete blood count, renal function tests, liver function tests, lipid profile, thyroid function tests, and glycemic parameters (plasma glucose and glycated hemoglobin). Efficacy was analyzed in 91 patients. ARE administration resulted in a significant reduction in body weight (-8.46 ± 3.86 kg; *P* < 0.0001) and BMI (-3.31 ± 1.57 kg/m^2^; *P* < 0.0001) compared to the PL (-2.41 ± 2.07 kg and -0.93 ± 0.79 kg/m^2^ for body weight and BMI, respectively). Compared with PL, ARE showed significant improvements (*P* < 0.05) in PSS, SF-12, SSS, and FCQ-T scores. A total of seven participants with ARE and six with PL reported mild adverse events (nausea, abdominal pain, and drowsiness), which were resolved without any intervention. Ashwagandha root extract may offer a safe and beneficial approach for stress reduction and weight management.

## INTRODUCTION

Stress is defined as any external or internal stimulus that disrupts the body’s internal equilibrium, known as homeostasis [1]. It plays a significant role in the pathophysiology of various chronic conditions, including depression, cardiovascular disease, diabetes, hypertension, and weight gain. Stress is often associated with symptoms such as headaches, fatigue, gastrointestinal disturbances, muscle tension, sleep disruptions, and cognitive dysfunction [2]. It is recognized as a key etiological factor contributing to weight gain [3].

Weight gain is a multifactorial condition influenced by lifestyle disorders and affects individuals of all ages and genders [4]. It results from a combination of genetic and physiological factors, excessive calorie intake, metabolic and respiratory dysfunction, glucose intolerance, environmental and social influences, and economic pressures [5].

Previous research has shown that stress induces weight gain, primarily through increased cortisol (a stress hormone) secretion [6]. Glucocorticoids are known to drive adipose tissue redistribution and visceral fat accumulation, while also increasing appetite and cravings for calorie-dense, palatable “comfort foods” rice, sugar, and fat [7,8]. These behaviours lead to elevated caloric intake and a higher body mass index (BMI) [9].

A cross-sectional study further demonstrated a significant association between chronic psychosocial stress, increased food cravings, and elevated BMI [10]. The mechanism linking stress to overeating and excessive weight gain is largely mediated by dysregulation of the hypothalamic–pituitary–adrenal (HPA) axis [11]. Activation of the HPA axis triggers cortisol release, which stimulates appetite and promotes the consumption of energy-dense foods. Chronic activation of this pathway leads to persistent appetite stimulation, uncontrollable eating habits, and progressive weight gain [12].

Several studies have investigated the effects of various herbal extracts traditionally recognized for their anxiolytic, adaptogenic, and metabolic properties on anxiety, stress, and body weight regulation [2, 13, 14]. These interventions have demonstrated reductions in both body weight and serum cortisol levels [13]. Among these, Ashwagandha (*Withania somnifera*) has been widely used in traditional medicine for its stress-relieving and adaptogenic effects.

Ashwagandha is a well-known Ayurvedic *Rasayana* herb traditionally employed to enhance cognitive function and memory through its adaptogenic and anti-stress effects. As an adaptogen, it helps to promote energy balance, rejuvenation, and revitalization [15]. The available preclinical and clinical studies have documented a broad spectrum of Ashwagandha’s physiological activities, including immunomodulatory [16,17], anti-inflammatory [18], antioxidant [19,20], and stress-reducing effects [16,21].

As a natural antioxidant, Ashwagandha contributes to overall health [17,21] by regulating metabolism, reducing inflammation, and supporting weight management [18,19]. Its antioxidant activity also strengthens immune function. Clinical studies have shown that Ashwagandha root extract improves mental well-being, eating behavior, and stress in overweight and obese adults experiencing chronic stress [14]. Preclinical evidence further indicates that Ashwagandha root extract reduces hippocampal neuron damage in the CA2 and CA3 regions by 80% [22].

In addition, Ashwagandha has been reported to lower serum cortisol, improve metabolic parameters, relieve psychological and physical stress, and simultaneously reduce food cravings and stress-induced eating behaviors [14]. It may also enhance energy metabolism, facilitating the utilization of fat rather than its storage [23]. This synergistic interplay of stress modulation, metabolic enhancement, and antioxidant activity underlies its potential for supporting healthy weight management.

Considering these multifaceted benefits, the present study was designed to evaluate the efficacy and safety of Ashwagandha Root Extract (ARE) in managing physiological stress and promoting weight loss in adults over a 24-week intervention period.

## MATERIAL AND METHODS

### Study design

This was a prospective, randomized, single-center, double-blind, placebo-controlled clinical trial designed to evaluate the efficacy and safety of ARE for managing stress and weight loss in overweight adults for a 24-week intervention period. The study was conducted at METABOL - Lifestyle Clinic for Metabolic Syndrome, Mumbai, India. The enrollment of participants commenced on May 15, 2023, and the study was completed on September 27, 2024. Written informed consent was obtained from all participants prior to the initiation of any study-related procedures.

### Study participants

**Inclusion criteria:** Adults aged 19 to 65 years with a BMI between 25.0 and 39.9 kg/m^2^ were eligible. Participants who were willing to sign written informed consent before trial commencement and agreed to take the investigational product for the full 24-week study duration were included.

**Exclusion criteria:** Individuals with a history of alcohol or smoking intake, hypersensitivity to study treatments, use of nutritional or energy supplements, medication, steroids, drug abuse, or clinical abnormalities were excluded from the study. Participants with known cardiovascular disease, diabetes mellitus, cerebrovascular disorders, clinically acute unstable hepatic, renal, respiratory, or any other neurological disorders, including depression, and mental health disorders, were excluded. Participants who used medication for blood pressure, beta-blockers, inhaled any beta-agonists, used any hormonal contraceptives, had a history of corticosteroid use within the last three months, or were under psychotropic medication within the last 8 weeks were not included. Additional exclusions included participants with an established meditation practice (>12 weeks), prior involvement in any clinical trial within the last 24 weeks, pregnancy, lactation, or any condition deemed unsafe by the study investigator.

### Sample size

Sample size calculations were based on a previously published randomized controlled trial by Chaudhary *et al*. [14] investigating the effects of ARE on stress and anxiety. The reported mean (standard deviation; SD) changes in BMI (kg/m^2^) were -2.32 (1.99) with the ARE group and -1.13 (1.24) with the placebo (PL) group. To detect a mean difference of 1.2 units in BMI between groups with SDs of 2.0 and 1.2, respectively, a minimum of 41 participants per group was required to achieve >90% power at a two-sided significant level (α) of 0.05, using an independent two-sample *t*-test. The study was powered to detect a clinically meaningful difference of 1.2 in body weight reduction, with a significance level of 5% and a power of 90%. Assuming a 10% data loss, it was planned to enrol 100 participants (50 in each arm) to achieve a final sample of 82 evaluable cases.

### Randomization and blinding

Participants were randomly assigned in a 1:1 ratio to receive either a capsule containing ARE (*n* = 50) or a placebo (PL; *n* = 50). The randomization sequence was generated using PC-based Rando version 1.2 software. Both the investigational and control products were identical in appearance, packaging, and labeling to maintain blinding. Upon enrollment, each participant was assigned a unique serial number corresponding to the pre-labeled medication pack. Allocation concealment was ensured by sequentially numbered, opaque, sealed envelopes that were opened only after participant enrollment. Investigators, study coordinators, and all personnel involved in participant assessment, data collection, and statistical analysis remained blinded to treatment allocation throughout the study. Unblinding was permitted only in the event of a medical emergency, following consultation with the principal investigator, and all instances and reasons for unblinding were documented in the study records.

### Interventions

Participants randomized to the ARE group received capsules containing 300 mg of standardized ARE (Ixoreal Biomed, CA, USA), and participants in the PL group received identical capsules containing 300 mg of inert starch. All participants were instructed to administer one capsule twice daily, after breakfast and after dinner, with a glass of water for 24 weeks. Throughout the study period, participants were advised to maintain their usual dietary habits and physical activity levels without implementing any specific lifestyle modifications.

#### Investigational products

The investigational product, standardized Ashwagandha root extract (KSM-66 Ashwagandha®), is a root-only extract manufactured using a green chemistry method (aqueous-based extraction process) that is devoid of alcohol or chemical solvents. The extract contains >5% withanolides, quantified by high-performance liquid chromatography (HPLC), with a drug-to-extract ratio of 12:1. The product is a light yellowish powder with a neutral taste.

### Study outcomes

The study was conducted for 24 weeks (180 days), during which participants attended four scheduled on-site visits: Screening/Baseline (Day 1), Week 4, Week 12, and End of study (Week 24). At baseline, participants were thoroughly evaluated for demographic data, medical and surgical history, and current medications. Efficacy and safety assessments were conducted at all scheduled visits, whereas the laboratory investigations were performed at baseline and week 24. Any adverse events (AEs) and concomitant medications were continuously monitored throughout the study period.

### Efficacy assessments

#### Primary outcomes

The mean change in body weight and BMI was recorded at Visit 1 (Screening/Enrolment/Baseline - Day 1), Visit 2 (Week 4), Visit 3 (Week 12), and Visit 4 (End of study - Week 24) to evaluate changes in overweight participants.

#### Secondary outcomes

The secondary outcomes including stress measured by the Perceived Stress Scale-10 (PSS-10), quality of life assessed using the Short Form-12 Health Survey (SF-12), Subjective Satisfaction Scale (SSS), and food craving assessed using the with Food Cravings Questionnaire-Trait (FCQ-T) were recorded at Visit 1 (Screening/Enrolment/Baseline - Day 1), Visit 2 (Week 4), Visit 3 (Week 12), and Visit 4 (End of study - Week 24).

#### Perceived Stress Scale

PSS-10 is a widely used, validated psychological instrument for measuring stress perception in individuals [24]. It evaluates the degree to which situations in one’s life were considered stressful, focusing on how unpredictable, uncontrollable, and overloaded participants found their lives to be over the past month. The scale comprises 10 items, each rated on a 5-point Likert scale ranging from 0 ("Never") to 4 ("Very often"). Four positively stated items (4, 5, 7, and 8) are reverse-scored. Total scores range from 0 to 40, with higher scores indicating greater perceived stress. Scores between 0 and 13 indicate low stress, 14 and 26 moderate stress, and 27 and 40 high perceived stress. The PSS-10 has demonstrated good internal consistency and reliability across diverse populations [25].

#### SF-12 Health Survey

SF-12 is a standardized, validated survey used to measure health-related quality of life in physical and mental health domains. It consists of 12 items from 8 health domains: physical functioning, physical role, bodily pain, general health, vitality, social functioning, emotional role, and mental health. The SF-12 has the Physical Component Summary (PCS) and the Mental Component Summary (MCS). The SF-12 is widely used in clinical trials due to its psychometric validity in both general and patient populations [26].

#### SSS

SSS is used to measure stress in various life contexts (e.g., well-being, including physical appearance, emotional balance, energy levels, and overall lifestyle changes) across different age groups and living conditions. It consists of 35 items, each answered using a 5-point Likert scale (1 = not at all, 2 = very little, 3 = neutral, 4 = somewhat, 5 = to a great extent) [27]. Higher scores indicate greater subjective satisfaction.

#### FCQ-T

The FCQ–T is used to assess the effects of study treatments on the frequency and intensity of food cravings. It consisted of 39 items scored on a 6-point scale ranging from never to always. Its original form comprises nine subscales measuring food cravings as intentions to consume food, anticipation of positive reinforcement, relief from negative states, lack of control over eating, preoccupation with food, hunger, emotions, cues that trigger cravings, and guilt [28].

### Safety assessments

Safety outcomes were based on treatment-emergent adverse events (TEAE) and laboratory investigations of blood samples.

#### Laboratory parameters

The laboratory parameters were complete blood count (CBC) and hemoglobin, lipid profile (total cholesterol, high-density lipoproteins, low-density lipoproteins, and triglycerides), liver function tests (LFT) such as serum aminase levels (aspartate and alanine), alkaline phosphatase, bilirubin (total and direct), serum cortisol, and plasma proteins (total proteins and albumin). Effects of interventions on the kidneys were assessed using serum creatinine, blood urea nitrogen, and uric acid, whereas serum T4 (thyroxine) and T3 (triiodothyronine) levels were evaluated for effects on the thyroid. Fasting plasma glucose and HbA1c levels were estimated. All blood laboratory tests were performed using a chemiluminescent immunoassay (CLIA). For serum cortisol, blood samples were collected from each participant in the morning via venipuncture. These levels were recorded at baseline, at week 4, week 12, and at week 24 of the study.

#### Adverse events

Throughout the 24-week treatment period, participants were monitored for any adverse events (AEs). They were instructed to report any unusual symptoms or side effects regularly. All reported AEs were categorized by severity, duration, and potential relationship to the study intervention. The monitoring process included weekly check-ins, physical exams, and laboratory tests to promptly identify and address any adverse reactions. This data helped ensure that any risks associated with Ashwagandha supplementation were identified, contributing to a thorough safety assessment and establishing the overall tolerability and risk-benefit profile of the treatment.

### Statistical methods and data analysis

Statistical data analysis was performed with Stata 13.1 (StataCorp LLC, USA). Descriptives were presented for all measurement data and scores of scales, whereas nominal data were presented as numbers and proportions. All analyses were conducted using two-sided tests at a 95% confidence level (cut-off value of 0.05 for *P*). A Chi-square test was used to compare the nominal data between two groups, whereas a two-sample *t*-test was used to compare measurement data and scores between the two groups. A General Linear Model (GLM) analysis was used to examine between-group and within-group effects, with treatment as the main factor and time as the repeated-measures factor.

**Figure 1:**
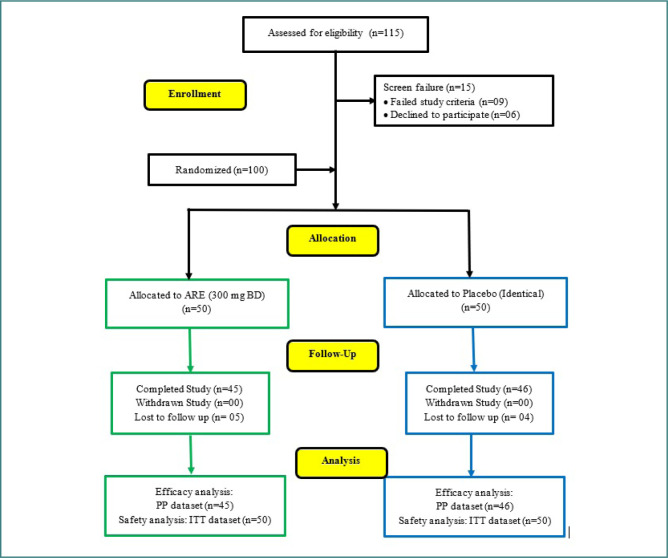
CONSORT flow chart

## RESULTS

A total of 115 adults were screened for eligibility. Of these, 15 participants were excluded due to screening failures: 9 failed to meet study inclusion criteria, and 6 declined to participate. Finally, 100 participants (adult men and women) meeting the eligibility requirements were enrolled in the study. During the study, 5 patients from the ARE and 4 from the PL group were lost to follow-up. The final efficacy analysis included 91 patients, 45 from the ARE group and 46 from the PL group (Figure 1).

### Demographic profile of patients and baseline data in the ITT dataset

Demographic features and baseline characteristics of participants enrolled in the study were comparable between the groups at enrollment, indicating successful randomization (Table 1).

### Primary outcome

**Figure 2:**
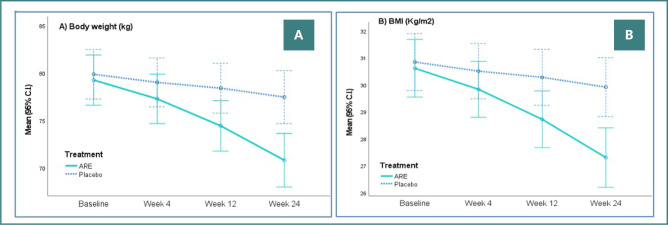
Body weight and BMI in the per-protocol (PP) dataset. Effect of ARE and placebo on: A, body weight (kg) and B, body mass index (BMI; kg/m^2^) over 24 weeks

At baseline, there were no statistically significant differences between the two groups in body weight (*P* = 0.922) or BMI (*P* = 0.875). However, significantly greater reductions in body weight and BMI were observed at weeks 4, 12, and 24 (*P* < 0.0001) with ARE compared to PL. At week 24, the mean BMI decreased by 3.31 kg/m^2^ in the ARE group compared to a reduction of only 0.93 kg/m^2^ with PL. These differences were statistically significant (*P* < 0.0001) with large effect sizes (Cohen’s d = –1.919). These values are summarized in Table 2 and Figure 2. Adjusted mean scores were calculated for body weight and BMI using the General Linear Model (GLM) procedure. The repeated-measures ANOVA revealed that both groups had reductions in body weight and BMI by week 24; however, the changes were greater in the ARE group.

### Secondary outcomes

**Figure 3:**
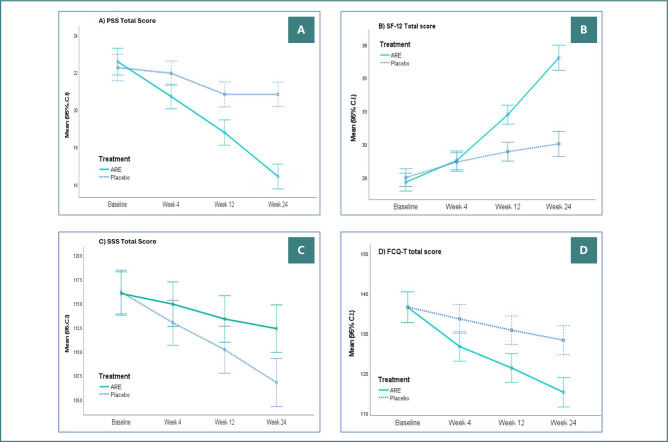
Total PSS, SF-12, SSS, and FCQ-T scores in the per-protocol (PP) dataset. Changes in psychological and behavioral outcome measures over time are shown as mean (95% CI) for: A, Perceived Stress Scale (PSS); B, Short Form-12 Quality of Life (SF-12); C, Subjective Satisfaction Scale (SSS); D, Food Craving Questionnaire–Trait (FCQ-T) in participants receiving ARE or placebo. The ARE group demonstrated progressive improvement in stress reduction, quality of life, and control of food cravings compared with placebo during the 24-week intervention.

The PSS, SF-12, and SSS scores were similar in the two groups at baseline (*P* > 0.05). There was greater improvement in all scores from baseline to week 24 with ARE compared with PL (Table 2, Figure 3A-C). Similarly, there was greater improvement (*P* < 0.0001) in the FCQ-T total scores with the ARE group (Table 3, Figure 3D).

The PSS-10 scores showed a mean reduction of -6.16 at week 24 in the ARE group, compared with -1.43 in the PL group (*P* < 0.001). Similarly, quality of life, measured by SF-12, increased by 9.38 points with ARE compared with 2.54 points with the PL group (*P* < 0.001). SSS decreased significantly in the ARE group (-9.42) than in PL (-3.65) by week 24 (*P* < 0.0001).

Significant differences were observed for the domains plan (*P* = 0.018), positive reinforcement (*P* = 0.001), negative reinforcement (*P* = 0.001), lack of control (*P* < 0.0001), hunger (*P* < 0.001), emotions (*P* < 0.0001), environment (*P* < 0.0001), guilt (*P* = 0.025) at week 24. The only domain where the difference was not statistically significant was ‘thoughts’ (*P* = 0.069). Overall, the ARE group showed key benefits over the PL in lowering the frequency and intensity of food cravings. Trait food cravings (FCQ-T) were markedly reduced in the ARE group (-21.24) compared to PL (-8.28) at the end of the study (*P* < 0.0001).

**Table 1:** Baseline data and profile of participants in the ITT dataset at baseline

	ARE (*n* = 50)	Placebo (*n* = 50)	
	Mean (SD)	Mean (SD)	P*
**Demography**			
Age (Years)	41.48 (10.12)	43.88 (8.88)	0.211
Weight (Kg)	79.73 (9.06)	79.90 (8.54)	0.922
BMI (Kg/m^2^)	30.69 (3.89)	30.80 (3.23)	0.875
**Laboratory values**			
FBS (mg/dL)	79.81 (15.69)	78.78 (19.7)	0.774
HbA1c (%)	4.49 (0.38)	4.85 (1.19)	0.046
Serum Cortisol (mcg/dL)	13.64 (2.67)	13.21 (3.85)	0.516
Total Cholesterol (mg/dL)	114.8 (29.17)	116.48 (28.18)	0.770
HDL-C (mg/dL)	43.42 (4.8)	42.46 (7.9)	0.463
LDL-C (mg/dL)	62.01 (10.92)	62.71 (15.25)	0.793
Triglycerides (mg/dL)	115.59 (22.64)	117.19 (27.24)	0.749
TSH (mIU/L)	4.63 (2.75)	4.69 (2.43)	0.911
T3 (ng/dL)	131.19 (22.34)	129.63 (26.31)	0.751
T4 (µg/dL)	8.28 (2.04)	7.80 (2.31)	0.272
	No. (%)	No. (%)	p**
**Gender**			
Male	21 (42.00%)	26 (52.00%)	0.316
Female	29 (58.00%)	24 (48.00%)	
**Comorbidity**			
Hypertension	2 (4.00%)	5 (10.00%)	0.576
Diabetes mellitus	3 (6.00%)	4 (8.00%)	
Thyroid disorders	0 (0.00%)	1 (2.00%)	
Total patients with comorbidity	5 (10.00%)	10 (20.00%)	
**Concomitant medications**			
Metformin	2 (4.00%)	2 (4.00%)	0.891
Telmisartan	2 (4.00%)	3 (6.00%)	
Teneligliptin	1 (2.00%)	1 (2.00%)	
Sitagliptin	0 (0.00%)	1 (2.00%)	
Total patients	5 (10.00%)	7 (14.00%)	

ARE, Ashwagandha Root Extract; BMI, Body Mass Index; FBS, Fasting Blood Sugar; HbA1c, Hemoglobin A1c; HDL-C, High-Density Lipoprotein Cholesterol; Kg, Kilogram; Kg/m^2^, Kilogram per Square Meter; LDL-C, Low-Density Lipoprotein Cholesterol; mg/dL, Milligrams per Decilitres; mIU/L, Milli-International Units per Liter; n, Number of patients; SD, Standard Deviation; yrs., Years; Min., Minimum; Max, Maximum; T3, Triiodothyronine; T4, Thyroxine; TSH, Thyroid-Stimulating Hormone; µg/dL, Micrograms per Deciliter. P*: two-sample *t*-test; P**: Chi-square test.

**Table 2:** Body weight, BMI, and total scores for PSS, SF-12, and SSS in two groups in the PP dataset

	ARE (*n* = 45)	Placebo (*n* = 46)	Difference	Effect size	*t*-test	
	Mean (SD)	Mean (SD)	Mean (95% C.I.)	Cohen’s ‘d’	*t*	P*
**Body weight (kg)**						
Baseline	79.23 (9.18)	79.85 (8.69)	-0.61 (-4.34 to 3.11)	-0.069	-0.327	0.745
Change at week 4	-1.99 (1.55)	-0.86 (1.05)	-1.13 (-1.68 to -0.58)	-0.856	-4.081	<0.0001
Change at week 12	-4.84 (3.22)	-1.47 (1.62)	-3.37 (-4.43 to -2.31)	-1.327	-6.329	<0.0001
Change at week 24	-8.46 (3.86)	-2.41 (2.07)	-6.05 (-7.34 to -4.77)	-1.960	-9.349	<0.0001
**BMI (Kg/m^2^)**						
Baseline	30.61 (3.14)	30.84 (4.00)	-0.23 (-1.73 to 1.27)	-0.064	-0.306	0.760
Change at week 4	-0.78 (0.62)	-0.34 (0.41)	-0.44 (-0.66 to -0.22)	-0.840	-4.006	<0.0001
Change at week 12	-1.89 (1.29)	-0.57 (0.63)	-1.33 (-1.75 to -0.90)	-1.307	-6.234	<0.0001
Change at week 24	-3.31 (1.57)	-0.93 (0.79)	-2.38 (-2.89 to -1.86)	-1.919	-9.152	<0.0001
**PSS Total Score**						
Baseline	22.58 (2.54)	22.26 (2.28)	0.32 (-0.69 to 1.32)	0.131	0.625	0.533
Change at week 4	-1.87 (1.69)	-0.30 (1.36)	-1.56 (-2.20 to -0.92)	-1.020	-4.863	<0.001
Change at week 12	-3.80 (2.25)	-1.43 (2.48)	-2.37 (-3.35 to -1.38)	-0.997	-4.757	<0.001
Change at week 24	-6.16 (1.52)	-1.43 (2.74)	-4.72 (-5.65 to -3.80)	-2.125	-10.135	<0.001
**SF-12 Total Score**						
Baseline	27.22 (2.41)	27.34 (2.20)	-0.12 (-1.04 to 0.80)	-0.052	-0.260	0.795
Change at week 4	1.64 (1.13)	1.17 (0.38)	0.47 (0.12 to 0.82)	0.560	2.669	0.009
Change at week 12	5.09 (1.31)	1.96 (0.59)	3.13 (2.71 to 3.55)	3.088	14.729	<0.001
Change at week 24	9.38 (2.71)	2.54 (1.11)	6.83 (5.98 to 7.69)	3.316	15.817	<0.001
**SSS total Score**						
Baseline	116.18 (9.15)	116.02 (5.87)	0.16 (-3.04 to 3.35)	0.020	0.097	0.923
Change at week 4	-3.20 (2.87)	-1.11 (0.95)	-2.09 (-2.98 to -1.20)	-0.982	-4.684	<0.0001
Change at week 12	-6.00 (4.17)	-2.65 (2.34)	-3.35 (-4.75 to -1.94)	-0.994	-4.739	<0.0001
Change at week 24	-9.42 (4.56)	-3.65 (2.46)	-5.77 (-7.29 to -4.25)	-1.581	-7.541	<0.0001

ARE, Ashwagandha Root Extract; BMI, Body Mass Index; CI, Confidence Interval; Kg, Kilogram; Kg/m^2^, Kilogram per Square Meter; *n*, Number of patients. PSS, Perceived Stress Scale; SD, Standard Deviation; SF-12, Short Form Health Survey (12-Item Version); SSS, Standard Stress Scale. *P < 0.05 suggests significant differences between the groups, and *P* > 0.05 suggests no significant differences between the groups.

**Table 3:** The FCQ-T scores in two groups in the PP dataset

	ARE (*n* = 45)	Placebo (*n* = 46)	Difference	Effect size	*t*-test	
	Mean (SD)	Mean (SD)	Mean (95% C.I.)	Cohen’s ‘d’	*t*	P*
**Plan**						
Baseline	9.96 (2.01)	9.91 (2.05)	0.04 (-0.80 to 0.89)	0.021	0.100	0.921
Change at week 4	-0.58 (1.91)	-0.17 (1.43)	-0.40 (-1.11 to 0.30)	-0.239	-1.141	0.257
Change at week 12	-0.82 (2.11)	-0.48 (1.43)	-0.34 (-1.09 to 0.41)	-0.191	-0.912	0.364
Change at week 24	-1.53 (2.56)	-0.43 (1.71)	-1.10 (-2.00 to -0.19)	-0.505	-2.410	0.018
**Positive Reinforcement**						
Baseline	17.84 (3.60)	17.87 (2.52)	-0.03 (-1.32 to 1.27)	-0.008	-0.039	0.969
Change at week 4	-0.58 (2.80)	-0.30 (0.79)	-0.27 (-1.13 to 0.58)	-0.134	-0.637	0.526
Change at week 12	-1.62 (2.79)	-0.63 (1.04)	-0.99 (-1.87 to -0.12)	-0.473	-2.256	0.027
Change at week 24	-2.44 (2.88)	-0.78 (1.21)	-1.66 (-2.58 to -0.75)	-0.755	-3.602	0.001
**Negative Reinforcement**						
Baseline	10.31 (2.95)	10.30 (2.59)	0.01 (-1.15 to 1.16)	0.002	0.012	0.991
Change at week 4	-1.27 (1.36)	-0.24 (0.71)	-1.03 (-1.48 to -0.58)	-0.954	-4.552	<0.0001
Change at week 12	-1.67 (1.77)	-0.63 (0.85)	-1.04 (-1.61 to -0.46)	-0.748	-3.568	0.001
Change at week 24	-2.27 (1.85)	-1.24 (0.82)	-1.03 (-1.62 to -0.43)	-0.720	-3.435	0.001
**Lack of control**						
Baseline	20.84 (3.34)	20.87 (2.58)	-0.03 (-1.27 to 1.22)	-0.008	-0.040	0.968
Change at week 4	-1.40 (2.05)	-0.50 (0.94)	-0.90 (-1.56 to -0.24)	-0.567	-2.704	0.008
Change at week 12	-2.00 (2.26)	-0.48 (1.24)	-1.52 (-2.28 to -0.77)	-0.838	-3.997	<0.0001
Change at week 24	-3.09 (2.36)	-0.85 (1.37)	-2.24 (-3.04 to -1.44)	-1.165	-5.554	<0.0001
**Thoughts**						
Baseline	24.27 (3.81)	24.26 (3.51)	0.01 (-1.52 to 1.53)	0.002	0.008	0.994
Change at week 4	-1.44 (1.96)	-0.26 (0.98)	-1.18 (-1.83 to -0.54)	-0.767	-3.658	<0.0001
Change at week 12	-1.98 (2.46)	-0.74 (1.14)	-1.24 (-2.04 to -0.44)	-0.647	-3.087	0.003
Change at week 24	-2.04 (3.53)	-1.04 (1.05)	-1.00 (-2.08 to 0.08)	-0.386	-1.842	0.069
**Hunger**						
Baseline	13.44 (2.46)	13.46 (1.95)	-0.01 (-0.94 to 0.91)	-0.005	-0.026	0.979
Change at week 4	-0.96 (1.41)	0.17 (1.39)	-1.13 (-1.71 to -0.55)	-0.807	-3.847	<0.0001
Change at week 12	-1.84 (1.35)	-0.37 (1.24)	-1.47 (-2.01 to -0.94)	-1.141	-5.443	<0.0001
Change at week 24	-2.58 (1.74)	-1.02 (1.36)	-1.56 (-2.20 to -0.91)	-0.999	-4.764	<0.0001
**Emotions**						
Baseline	14.82 (3.03)	14.83 (2.73)	0.00 (-1.21 to 1.20)	-0.001	-0.006	0.995
Change at week 4	-1.42 (2.99)	-0.46 (1.26)	-0.97 (-1.92 to -0.01)	-0.423	-2.016	0.047
Change at week 12	-1.93 (2.37)	-0.76 (0.95)	-1.17 (-1.92 to -0.42)	-0.653	-3.113	0.002
Change at week 24	-3.07 (2.36)	-1.22 (1.19)	-1.85 (-2.63 to -1.07)	-0.993	-4.736	<0.0001
**Environment**						
Baseline	13.87 (2.40)	13.87 (1.80)	0.00 (-0.88 to 0.88)	-0.001	-0.007	0.995
Change at week 4	-0.78 (2.18)	-0.67 (1.40)	-0.10 (-0.87 to 0.66)	-0.057	-0.271	0.787
Change at week 12	-1.62 (1.54)	-0.87 (1.20)	-0.75 (-1.33 to -0.18)	-0.545	-2.599	0.011
Change at week 24	-2.38 (2.39)	-0.85 (1.35)	-1.53 (-2.34 to -0.72)	-0.792	-3.775	<0.0001
**Guilt**						
Baseline	11.11 (2.85)	11.15 (2.94)	-0.04 (-1.25 to 1.17)	-0.014	-0.068	0.946
Change at week 4	-1.38 (2.71)	-0.35 (1.32)	-1.03 (-1.91 to -0.15)	-0.485	-2.314	0.023
Change at week 12	-1.67 (2.80)	-0.80 (1.53)	-0.86 (-1.80 to 0.07)	-0.384	-1.831	0.071
Change at week 24	-1.84 (2.54)	-0.85 (1.51)	-1.00 (-1.86 to -0.13)	-0.479	-2.283	0.025
**FCQ-T total score**						
Baseline	136.47 (14.68)	136.52 (11.27)	-0.06 (-5.50 to 5.39)	-0.004	-0.020	0.984
Change at week 4	-9.80 (6.50)	-2.96 (3.81)	-6.84 (-9.06 to -4.63)	-1.288	-6.145	<0.0001
Change at week 12	-15.16 (7.50)	-5.76 (4.03)	-9.39 (-11.90 to -6.89)	-1.565	-7.463	<0.0001
Change at week 24	-21.24 (7.24)	-8.28 (3.90)	-12.96 (-15.38 to -10.55)	-2.236	-10.663	<0.0001

ARE, Ashwagandha Root Extract; C.I., Confidence interval; FCQ-T, Food Craving Questionnaire-Trait; *N*, Patients count; SEM, Standard error mean; SD, Standard deviation; Min., Minimum; Max, Maximum. *P < 0.05 suggests significant differences between the groups, and *P* > 0.05 suggests no significant differences between the groups.

**Table 4:** Laboratory parameters in two groups in the PP dataset

		ARE (*n* = 45)	Placebo (*n* = 46)	*t*-test	
		Mean (SD)	Mean (SD)	*t*	*P*
**Blood counts**					
WBC Count (X100 cells/µL)	Baseline	71.68 (14.11)	71.35 (10.66)	0.128	0.899
	Week 4	73.79 (13.69)	75.19 (10.70)	-0.544	0.588
	Week 12	79.96 (14.08)	77.53 (9.49)	0.970	0.334
	Week 24	78.30 (15.18)	80.06 (11.06)	-0.633	0.528
RBC Count (million cells/µL)	Baseline	4.97 (0.44)	4.99 (0.42)	-0.212	0.832
	Week 4	5.02 (0.53)	5.03 (0.50)	-0.076	0.940
	Week 12	5.07 (0.45)	5.04 (0.43)	0.337	0.737
	Week 24	5.09 (0.53)	5.07 (0.62)	0.157	0.876
Haematocrit (%)	Baseline	40.48 (2.35)	40.54 (2.56)	-0.119	0.905
	Week 4	41.18 (2.08)	40.85 (2.91)	0.619	0.538
	Week 12	41.38 (2.21)	41.20 (2.38)	0.372	0.711
	Week 24	41.69 (2.32)	41.70 (2.73)	-0.024	0.981
Hb (gm/dL)	Baseline	13.51 (1.39)	13.42 (0.91)	0.438	0.662
	Week 4	14.06 (1.08)	13.37 (0.94)	2.964	0.004
	Week 12	14.38 (1.11)	13.33 (1.01)	4.805	<0.001
	Week 24	14.51 (0.98)	13.44 (1.06)	5.485	<0.001
Platelet count (X10000 cells/µL)	Baseline	25.71 (5.77)	24.35 (6.88)	1.021	0.310
	Week 4	26.06 (5.74)	27.51 (7.26)	-1.062	0.291
	Week 12	27.53 (7.97)	28.90 (9.04)	-0.765	0.446
	Week 24	26.50 (6.10)	27.71 (7.85)	-0.816	0.417
**Glycemic parameters**					
FBS (mg/dL)	Baseline	77.52 (11.67)	78.74 (20.45)	-0.350	0.727
	Week 4	83.04 (16.51)	85.71 (16.74)	-0.766	0.446
	Week 12	91.24 (10.75)	94.33 (15.25)	-1.112	0.269
	Week 24	94.98 (12.94)	94.20 (16.54)	0.251	0.802
HbA1c (%)	Baseline	4.48 (0.39)	4.88 (1.23)	-2.080	0.040
	Week 4	6.09 (9.60)	4.82 (0.75)	0.900	0.370
	Week 12	4.74 (0.57)	5.08 (0.85)	-2.236	0.028
	Week 24	4.58 (0.61)	4.62 (0.65)	-0.349	0.728
**Hepatic function tests**					
Total Bilirubin (mg/dL)	Baseline	0.60 (0.54)	0.65 (0.65)	-0.460	0.647
	Week 4	0.57 (0.32)	0.69 (1.00)	-0.758	0.450
	Week 12	0.49 (0.21)	0.50 (0.25)	-0.226	0.822
	Week 24	0.63 (1.38)	0.46 (0.16)	0.845	0.401
Direct Bilirubin (mg/dL)	Baseline	0.31 (0.42)	0.28 (0.26)	0.352	0.726
	Week 4	0.36 (0.28)	0.30 (0.29)	1.072	0.287
	Week 12	0.24 (0.09)	0.28 (0.11)	-1.974	0.051
	Week 24	0.25 (0.09)	0.27 (0.09)	-1.038	0.302
Indirect Bilirubin (mg/dL)	Baseline	0.34 (0.21)	0.29 (0.29)	1.009	0.316
	Week 4	0.28 (0.29)	0.47 (1.34)	-0.918	0.361
	Week 12	0.24 (0.14)	0.22 (0.11)	0.741	0.461
	Week 24	0.19 (0.09)	0.19 (0.09)	0.043	0.966
Alkaline Phosphatase (IU/L)	Baseline	82.86 (28.61)	72.98 (17.26)	2.000	0.049
	Week 4	71.26 (24.94)	78.13 (21.64)	-1.404	0.164
	Week 12	76.81 (18.29)	78.41 (19.11)	-0.408	0.684
	Week 24	90.66 (16.82)	93.98 (20.35)	-0.847	0.399
AST (IU/L)	Baseline	23.12 (4.54)	23.50 (4.71)	-0.387	0.700
	Week 4	23.74 (7.57)	24.17 (4.45)	-0.331	0.742
	Week 12	21.94 (4.50)	23.80 (5.48)	-1.767	0.081
	Week 24	23.59 (6.02)	23.65 (5.38)	-0.053	0.958
ALT (IU/L)	Baseline	20.39 (13.35)	20.20 (7.24)	0.087	0.931
	Week 4	22.59 (14.64)	20.02 (6.84)	1.077	0.285
	Week 12	20.13 (5.01)	20.28 (6.48)	-0.123	0.903
	Week 24	21.75 (4.50)	20.54 (4.44)	1.287	0.202
Total Protein (g/dL)	Baseline	6.98 (0.90)	6.91 (1.15)	0.324	0.746
	Week 4	7.27 (2.13)	8.64 (12.00)	-0.753	0.453
	Week 12	6.90 (0.79)	7.36 (2.08)	-1.406	0.163
	Week 24	6.86 (0.85)	6.82 (0.70)	0.191	0.849
Albumin (g/dL)	Baseline	3.90 (0.89)	3.96 (0.89)	-0.369	0.713
	Week 4	3.80 (0.82)	4.14 (0.95)	-1.831	0.070
	Week 12	3.92 (0.83)	4.19 (0.95)	-1.401	0.165
	Week 24	3.48 (0.63)	3.83 (1.09)	-1.860	0.066
**Renal function tests**					
Creatinine (mg/dL)	Baseline	0.73 (0.15)	0.74 (0.20)	-0.159	0.874
	Week 4	0.92 (0.91)	0.72 (0.13)	1.418	0.160
	Week 12	0.78 (0.16)	0.77 (0.16)	0.368	0.714
	Week 24	0.79 (0.12)	0.75 (0.11)	1.748	0.084
Urea (mg/dL)	Baseline	13.25 (7.02)	11.80 (4.68)	1.163	0.248
	Week 4	15.45 (24.10)	12.02 (3.61)	0.957	0.341
	Week 12	12.20 (4.86)	13.21 (5.41)	-0.937	0.351
	Week 24	10.89 (4.74)	10.70 (3.00)	0.222	0.824
BUN (mg/dL)	Baseline	10.44 (4.90)	11.30 (5.39)	-0.794	0.429
	Week 4	10.52 (5.19)	10.16 (5.01)	0.328	0.744
	Week 12	10.45 (5.33)	12.32 (5.38)	-1.659	0.101
	Week 24	15.15 (5.62)	14.90 (5.45)	0.212	0.833
Uric Acid (mg/dL)	Baseline	6.03 (2.09)	6.03 (2.18)	0.007	0.994
	Week 4	5.72 (2.21)	5.76 (2.38)	-0.089	0.929
	Week 12	6.00 (2.10)	6.12 (2.08)	-0.272	0.786
	Week 24	6.66 (1.30)	6.20 (1.52)	1.527	0.130
Calcium (mg/dL)	Baseline	8.46 (0.40)	8.39 (0.32)	0.846	0.400
	Week 4	8.47 (0.30)	8.03 (1.50)	1.933	0.056
	Week 12	8.37 (0.29)	8.35 (0.30)	0.305	0.761
	Week 24	8.33 (0.27)	8.29 (0.23)	0.840	0.403
**Lipid profile**					
Cholesterol (mg/dL)	Baseline	116.43 (30.00)	116.77 (29.14)	-0.055	0.957
	Week 4	120.70 (29.53)	126.70 (30.40)	-0.955	0.342
	Week 12	114.78 (19.09)	127.02 (31.64)	-2.228	0.028
	Week 24	123.45 (32.30)	129.30 (33.56)	-0.848	0.399
HDL (mg/dL)	Baseline	43.80 (4.84)	42.52 (8.14)	0.911	0.365
	Week 4	45.36 (11.67)	44.80 (8.76)	0.260	0.796
	Week 12	44.91 (6.24)	47.99 (9.43)	-1.838	0.069
	Week 24	45.57 (9.64)	46.65 (10.04)	-0.523	0.602
LDL (mg/dL)	Baseline	62.11 (11.32)	62.66 (15.48)	-0.195	0.846
	Week 4	64.55 (13.42)	67.67 (13.31)	-1.112	0.269
	Week 12	65.25 (14.67)	69.97 (12.13)	-1.673	0.098
	Week 24	69.17 (18.50)	66.93 (11.15)	0.702	0.485
Triglycerides (mg/dL)	Baseline	115.48 (23.68)	116.86 (27.88)	-0.254	0.800
	Week 4	124.59 (30.05)	117.74 (25.79)	1.168	0.246
	Week 12	117.36 (28.85)	121.54 (20.33)	-0.801	0.425
	Week 24	120.36 (30.68)	123.76 (28.37)	-0.549	0.584
**Thyroid function tests**					
TSH (mIU/L)	Baseline	4.70 (2.83)	4.69 (2.42)	0.015	0.988
	Week 4	5.14 (2.55)	4.38 (2.47)	1.448	0.151
	Week 12	5.70 (3.44)	6.89 (10.14)	-0.745	0.458
	Week 24	4.29 (3.83)	4.17 (2.63)	0.186	0.853
T3 (ng/dL)	Baseline	131.39 (23.49)	128.73 (27.15)	0.498	0.619
	Week 4	134.88 (30.23)	128.10 (25.09)	1.165	0.247
	Week 12	135.19 (25.06)	132.62 (31.50)	0.429	0.669
	Week 24	132.15 (33.10)	132.04 (25.92)	0.017	0.987
T4 (µg/dL)	Baseline	8.29 (2.13)	7.84 (2.30)	0.965	0.337
	Week 4	10.34 (15.81)	10.23 (13.61)	0.035	0.972
	Week 12	7.88 (1.95)	8.32 (2.11)	-1.036	0.303
	Week 24	7.55 (2.76)	7.65 (2.42)	-0.200	0.842

ALP, Alkaline Phosphatase; ALT, Alanine Aminotransferase; AST, Aspartate Aminotransferase; BUN, Blood Urea Nitrogen; Ca, Calcium; Chol, Cholesterol; FBS, Fasting Blood Sugar; Hb, Hemoglobin; HbA1c, Glycated Hemoglobin; HDL, High-Density Lipoprotein; LDL, Low-Density Lipoprotein; RBC, Red Blood Cell Count; T3, Triiodothyronine; T4, Thyroxine; TSH, Thyroid-Stimulating Hormone; WBC, White Blood Cell Count; U/L, International Units per Liter; mg/dL, Milligrams per Decilitres; gm/dL, Grams per Decilitres; %, Percentage; X10000 cells/cmm, Cells per 10,000 Cubic Millimetres; cells/µL, Cells per Microliter; g/dL, Grams per Decilitres; ng/dL, Nanograms per Decilitres; µg/dL, Micrograms per Decilitres; mIU/L, Milli-International Units per Liter

**Table 5:** Serum cortisol levels in participants from baseline to week 24 in the PP dataset (*n* = 91)

Group Statistics	Groups	*n*	Mean	SD	Difference		95% C.I. for difference		Unpaired *t*-test		Effect Size	95% C.I. for ‘d’	
					Mean	SEM	Lower	Upper	*t*	*P*	Cohen’s d	Lower	Upper
**Sr. Cortisol (mcg/dL)**													
**Baseline**	ARE	45	13.91	2.20	0.15	0.60	-1.04	1.34	0.251	0.803	0.053	-0.359	0.463
	Placebo	46	13.76	3.38									
**Week 12**	ARE	45	11.45	2.05	-1.05	0.56	-2.17	0.06	-1.873	0.064	-0.393	-0.807	0.023
	Placebo	46	12.50	3.18									
**Week 24**	ARE	45	8.90	3.49	-2.32	0.82	-3.95	-0.70	-2.836	0.006	-0.595	-1.013	-0.173
	Placebo	46	11.22	4.27									
**Change at 12 wks.**	ARE	45	-2.47	1.98	-1.19	0.43	-2.04	-0.34	-2.792	0.006	-0.585	-1.004	-0.164
	Placebo	46	-1.28	2.09									
**Change at 24 wks.**	ARE	45	-5.02	3.91	-2.47	0.85	-4.16	-0.78	-2.908	0.005	-0.610	-1.029	-0.188
	Placebo	46	-2.55	4.20									

ARE, Ashwagandha Root Extract; C.I, Confidence interval; *n*, Patients count; SEM, Standard error mean; SD, Standard deviation; *P*, Unpaired *t*-test

The adjusted scores showed significant group effects for the PSS-10 (*P* = 0.007, observed power = 0.78), SF-12 (*P* = 0.033, observed power = 0.57), and FCQ-T (*P* = 0.005, observed power = 0.82).

During the 24-week treatment period, 13 participants reported mild adverse events (AEs), including seven from the ARE group and six from the placebo (PL) group. In both groups, nausea was reported by three participants each (6.0%), and abdominal pain by two participants each (4.0%). Drowsiness occurred in two participants (4.0%) from the ARE group and one participant (2.0%) from the PL group. Overall, seven participants (14.0%) in the ARE group and six participants (12.0%) in the PL group experienced AEs. All reported events were mild in intensity, required no medical intervention, and resolved spontaneously without any lasting effects.

The study found no significant differences between the ARE and PL groups in blood counts, hepatic parameters, renal parameters, lipid profile, and thyroid function tests at baseline, except serum uric acid (*P* = 0.042), which was not clinically significant. Post-treatment laboratory assessments in week 24 in the PP dataset did not show any clinically significant changes in any parameters in either group (*P* > 0.05; Table 4). However, in week 12, lower laboratory values were observed for ARE of HbA1c (*P* = 0.028) and serum total cholesterol (*P* = 0.028). These results suggest that ARE may have beneficial effects on glycemic control and lipid profiles.

Serum cortisol levels declined significantly in the ARE group compared to PL over the 24-week study period. At baseline, cortisol levels were comparable between groups (ARE: 13.91 ± 2.20 mcg/dL; PL: 13.76 ± 3.38 mcg/dL). By week 24, a significant reduction was reported in the ARE group (8.90 ± 3.49 mcg/dL) compared to the PL group (11.22 ± 4.27 mcg/dL), with a statistically significant between-group difference ( -2.32 mcg/dL, *P* = 0.006, Cohen’s d = -0.595) (Table 5). Similarly, the mean change from baseline to week 24 was significantly higher in the ARE group (-5.02 ± 3.91) compared to PL (-2.55 ± 4.20), with an effect size of -0.610 (*P* = 0.005).

## DISCUSSION

In this prospective, double-blind, randomized, placebo-controlled study, the efficacy and safety of ARE (300 mg twice daily) were assessed in adults for the management of stress and weight for 24 weeks. The 24-week study duration was selected to evaluate the long-term efficacy and safety of ARE, as previous clinical investigations have demonstrated favorable outcomes at shorter durations (8 weeks). Extending the treatment period allowed assessment of sustained effects on stress reduction and weight management, as well as the continued safety of ARE with prolonged use. The study objective was to assess changes in body weight and BMI, along with secondary outcomes including stress, quality of life, subjective satisfaction, and food cravings. The study findings demonstrated significant reductions in body weight and BMI. Also, there was a reduction in the levels of stress (PSS & SSS) and food cravings (FCQ-T) compared to PL, with an overall improvement in SF-12 scores.

Chronic stress is closely linked to unhealthy eating behaviors and weight gain [29,30]. Stress can alter metabolism and neuroendocrine responses, including elevated cortisol levels, reduced sleep quality, and disturbed appetite control. It triggers a systemic rise in cortisol, which contributes to increased visceral fat and other aspects of metabolic syndrome, such as obesity, hypertension, dyslipidemia, and type 2 diabetes [31,32]. Therefore, effective stress management promotes body weight and BMI reduction.

Preclinical evidence has consistently highlighted the neuroprotective, anti-obesity, and stress-mitigating effects of *Withania somnifera* (Ashwagandha) root extracts. Experimental studies have shown that supplementation with Ashwagandha roots ameliorates obesity-induced metabolic and neurological disturbances by modulating neurotransmitter levels, lipid metabolism, and oxidative stress markers. In an obese rat model, Ashwagandha root supplementation resulted in significant reductions in body weight gain, feed intake, and feed efficiency ratio, along with improvements in serum lipid profile and hepatic function. Furthermore, it elevated dopamine and serotonin levels and normalized acetylcholinesterase activity, thereby alleviating obesity-linked neurological impairments reported by Yousif *et al.* [33]. Another preclinical study by Jayshree *et al*. [34] demonstrated the anxiolytic and antidepressant effects of standardized ARE in a chronic stress rat model. ARE treatment improved behavioral parameters and favorably modulated biochemical markers, including elevated serotonin and brain-derived neurotrophic factor (BDNF) levels and reduced cortisol, adrenocorticotropic hormone (ACTH), corticotropin-releasing hormone (CRH), and proinflammatory cytokines, including interleukin-6 (IL-6) and tumor necrosis factor-alpha (TNF-α). These studies collectively provide mechanistic support for Ashwagandha’s adaptogenic and neuroregulatory actions through modulation of the hypothalamic–pituitary–adrenal (HPA) axis, neurotransmitter regulation, and antioxidant defense.

Consistent with these experimental observations, the present study demonstrated positive effects of ARE on stress and weight management. The weight loss observed in the ARE group suggests its potential as an effective intervention for weight management. In addition to being quantitative, this decrease represents better health outcomes, considering that overweight and obese people are more prone to developing a number of metabolic diseases. Similarly, a study conducted by Choudhary *et al.* [14] found that ARE had a positive impact on food cravings and weight management compared to PL. Treatment with the ARE led to clinically meaningful reductions in both perceived stress and serum cortisol across the current study and in Choudhary *et al.* [14]. In the 8-week trial (age: 18-60 years), PSS decreased by 32%, with cortisol reduced significantly (*P* = 0.0132), reflecting an early and strong effect [35]. In the current trial among adults (19-65 years), PSS declined by 27.28%, while cortisol dropped from 13.91 to 8.90 mcg/dL (−36%; *P* = 0.006), indicating slower but sustained benefits. These differences may relate to age-linked variations in HPA axis reactivity. The overall results are consistent across the two studies, although minor variations may be attributed to differences in age-related HPA axis reactivity. Collectively, these findings support both the short-term efficacy and long-term adaptogenic role of the extract in stress management. Participants in the ARE group showed statistically significant improvements in PSS (P <0.001) and SSS (P < 0.0001) scores, compared to PL, reflecting reduced stress-related symptoms. Also, the Ashwagandha group showed a significant reduction in PSS scores at both 4 (*P* = 0.001) and 12 weeks (*P* < 0.001), suggesting improvement compared with the PL group. It also reported a significant reduction in body weight and BMI, consistent with the present study. Another study with similar results was reported by Chandrasekhar *et al*. [2] after 60 days of ARE administration. The result demonstrated that the Ashwagandha group experienced a 44.0% reduction as compared to the PL group, which showed a 5.5% reduction in baseline PSS scores.

The SF-12 score is a measure of the impact of health on an individual’s everyday life, often used as a quality-of-life measure. The present study showed improvement (*P* < 0.0001) in SF-12 scores, suggesting better overall mental and physical health after ARE administration compared with PL.

The FCQ–T scale is a common tool for evaluating the effects of a study intervention on the frequency and intensity of food cravings. The FCQ-T scores in the present study demonstrated substantial benefits in several aspects, with significant *P* values ranging from 0.001 to <0.0001. These outcomes suggest the dual benefits of ARE in reducing both weight and stress, which are often interlinked. Similarly, a study by Choudhary *et al.* [19] showed that mean FCQ scores for planning, positive reinforcement, negative reinforcement, lack of control, emotion, and environment significantly improved (*P* < 0.05) after 8 weeks of Ashwagandha root extract consumption. The scores for thoughts about food, physiological, and guilt did not show significant changes. In contrast, in the present study, the only aspect of the FCQ-T score that showed no statistically significant difference was “Thoughts” (*P* = 0.069). These results suggest that Ashwagandha’s anxiolytic and antistress properties helped participants avoid using food as a stress-coping mechanism. Stress-induced food cravings, which can lead to unconscious eating, were mitigated, but parameters like "Thoughts about Food" remained unaffected by the treatment. Previous research has strongly supported the potential of Ashwagandha as a natural treatment for reducing stress and anxiety.

The FCQ–T scale is a validated measure for assessing the frequency and intensity of food cravings. In the present study, FCQ–T scores demonstrated significant improvements across multiple domains (*P* = 0.001 to <0.0001), highlighting the dual impact of ARE in alleviating both stress and weight-related behaviors. Consistent with these findings, Choudhary *et al.* [19] reported significant reductions in several craving domains following eight weeks of Ashwagandha root extract supplementation, although “Thoughts about food,” “Physiological,” and “Guilt” domains remained unaffected. In the current study, only the “Thoughts” domain did not reach statistical significance (*P* = 0.069). This observation may be attributed to the relative stability of cognitive patterns associated with food, which are less amenable to short- or medium-term modulation compared to emotional or behavioral aspects. In contrast, Ashwagandha’s established anxiolytic and antistress properties appear to predominantly influence stress-induced and emotional eating behaviors rather than deeply ingrained cognitive tendencies toward food [14,36].

Serum cortisol is the most widely used biomarker for evaluating physiological stress. Hence, it was used in the present study to measure the effect of ARE on cortisol levels. In the prospective, randomized 8-week study evaluating the effect of ARE on adults with stress, the change in cortisol levels was significant (*P* = 0.002) [23]. Another 8-week study involving 60 healthy adults experiencing stress investigated the effects of ARE 125 mg BD, 300 mg BD, and PL. Cortisol levels were reduced from 22.65 (1.75) mcg/dl to 15.00 (2.21) mcg/dl at week 8 with ARE 125 mg BD, and from 22.95 (1.57) mcg/dl to 14.15 (2.62) mcg/dl, showing a significant reduction [16]. Similarly, in the present study, the ARE group demonstrated a mean change in serum cortisol level of -5.02, while the PL group exhibited a mean change of -2.55 (*P* = 0.005) after 24 weeks. This further confirms the long-term efficacy of the ARE intervention in reducing cortisol levels, supported by a moderate to large effect size (Cohen’s d = -0.610).

The present study reported TEAEs in 13 patients (seven from the ARE group and six from the PL group). According to the Common Terminology Criteria for Adverse Events, all reported AEs were categorized as mild (grade 1) [37]. The grade 1 AEs are defined as transient or mild discomfort that does not interfere with daily activities, does not require therapeutic intervention, and resolves spontaneously. Both groups exhibited the same incidence of nausea and abdominal pain. Drowsiness occurred slightly more frequently in the ARE group compared to the PL group. The absolute difference in total AE incidence between groups was minimal (2.0%), representing no clinically significant risk associated with ARE intake. All reported AEs were physiological responses, indicating adaptive changes in the gastrointestinal or central nervous system, but did not indicate any pathological change. No participant required medical intervention, and all symptoms resolved completely without sequelae. This pattern of mild, self-limiting, and non-pathological AEs supports the favorable safety profile of ARE over a 24-week administration period. No serious adverse events were reported in this study, indicating the safety of ARE use for 24 weeks. As part of safety assessments, various laboratory parameters were assessed. The study found no significant differences between the ARE and PL groups in complete blood count, hepatic function tests, renal function tests, lipid profile, and thyroid function tests at baseline, except for serum uric acid (*P* = 0.042), which was not clinically significant. Post-treatment laboratory assessments in week 24 in the PP dataset did not show any clinically significant changes in any parameters in either group (*P* > 0.05; Table 4). However, in week 12, the lower laboratory values were observed with ARE for HbA1c (*P* = 0.028) and serum total cholesterol (*P* = 0.028). These results suggest that ARE may have beneficial effects on glycemic control and lipid profiles. Based on these findings, the use of ARE for 24 weeks, with no clinically significant changes in laboratory parameters, suggests that Ashwagandha root extract can be safe for long-term use. Numerous studies have shown that long-term use of Ashwagandha root extract is safe [23,38,39,40].

The present study’s strength lies in a randomized controlled trial (RCT) design, which reduces bias and enhances reliability. The comprehensive analysis included a wide range of physical and psychological outcomes, supported by adequate statistical power, confirming the robustness of the study’s findings. In addition, no serious adverse events were reported, and no clinically significant changes in laboratory parameters were observed after 24 weeks of ARE use, indicating its safety for a long-term profile. However, there are some limitations, such as a single-center design, sample size, and limited demographics, which may affect the generalizability of the results. Furthermore, potential confounding factors such as diet, sleep quality, and physical activity levels were not monitored or controlled. These lifestyle variables could have influenced outcomes related to stress, body weight, and overall well-being, potentially introducing bias. Future studies with multiple sites and more demographics are recommended to confirm these findings and explore the long-term benefits of ARE.

## CONCLUSION

The present study demonstrates that Ashwagandha root extract significantly reduces stress levels, thereby contributing to decreased body weight and reduced food cravings. These effects were accompanied by improvements in multiple psychological and behavioral outcomes compared to the placebo. ARE was well tolerated, with no serious adverse events or clinically meaningful changes in laboratory safety parameters observed over 24 weeks. Overall, these findings support the long-term use of ARE as a safe and promising intervention for reducing stress and stress-related weight management, and improving overall physical and mental well-being in overweight adults.

## Data Availability

The data supporting the findings of this study are available from the corresponding author upon reasonable request.

## Personal thanks

The authors thank Ixoreal BioMed Inc., Los Angeles, California, USA, for supplying the KSM-66 Ashwagandha root extract used in the study. We would like to acknowledge all the study investigators for their contributions. We thank Clinsearch Healthcare Solutions Pvt. Ltd, Thane, Maharashtra, for writing and editorial support.
